# Analysis of Stator Material Influence on BLDC Motor Performance

**DOI:** 10.3390/ma18194630

**Published:** 2025-10-07

**Authors:** Daniel Ziemiański, Gabriela Chwalik-Pilszyk, Grzegorz Dudzik

**Affiliations:** Faculty of Mechanical Engineering, Cracow University of Technology, Jana Pawła II 37 Avenue, 31-864 Krakow, Poland

**Keywords:** ferromagnetic materials, BLDC motor, power electronic, FEMM analysis

## Abstract

Brushless DC (BLDC) motors are increasingly used in industrial applications due to their high efficiency, reliability, and low weight. However, their performance strongly depends on the electromagnetic properties of stator and rotor core materials. This study evaluates six BLDC motor configurations, employing materials such as M19 electrical steel, 1010 low-carbon steel, magnetic PLA, and ABS, and analyzes their impact using FEMM 4.2 finite element simulations. Key electromagnetic characteristics—including flux linkage, Back-EMF, torque, and torque ripple—were compared across configurations. The reference motor with M19 steel stator and 1010 steel rotor achieved ~7 mWb flux linkage, ~39 V pk–pk Back-EMF, and 1.44 Nm torque with ~49% ripple, confirming the suitability of laminated steels for high-power-density designs. Substituting M19 with 1010 steel in the stator reduced torque by less than 10%, indicating material interchangeability with minimal performance loss. By contrast, polymer-based designs exhibited drastic degradation: magnetic PLA yielded only 3.5% of the baseline torque with sixfold ripple increase, while ABS delivered nearly zero torque and >700% ripple. Hybrid configurations improved PLA-based results by 15–20%, though they remained far below ferromagnetic cores. Overall, results demonstrate a nearly linear relationship between material permeability and both flux linkage and Back-EMF, alongside a sharp rise in torque ripple at low permeability. The findings highlight the advantages of ferromagnetic and laminated steel cores for efficiency and stability, while polymer and hybrid cores are limited to lightweight demonstrator applications.

## 1. Introduction

Brushless Direct Current (BLDC) motors are extensively employed across a broad spectrum of engineering applications, including ventilation systems, pumps, actuators, and various household appliances such as washing machines, DVD players, and computer peripheral devices [1,2,3,4]. In contrast to conventional direct current motors, where the commutation process is mechanically executed via brushes and a commutator, BLDC motors utilize electronic commutation implemented through semiconductor power switches. The absence of brush-based components eliminates common operational issues associated with mechanical wear, thereby reducing maintenance demands, enhancing system reliability, and ensuring quieter drive operation. Moreover, the incorporation of permanent magnets in the rotor facilitates efficient magnetic flux generation, which, in turn, contributes to improved energy efficiency, a more compact structural design, and reduced acoustic emissions [1,2,5].

The electrical characteristics of a BLDC motor are comparable to those of a conventional direct current motor; however, their reliability is significantly enhanced through the replacement of mechanical commutation with electronic commutation. Among the advantages of this motor type are an extended speed range, superior dynamic response, reduced operational noise, and lower overall mass [6,7,8,9].

BLDC motors are frequently deployed in harsh operating environments, where they are capable of withstanding overload conditions and elevated temperatures [9]. According to Da et al. [10] and Shifat et al. [11], faults in BLDC motors can be classified into mechanical, electrical, and magnetic categories. Approximately 30–40% of BLDC motor faults occur within the stator [12,13,14,15]. The predominant stator-related faults involve insulation failures, which can lead to short circuits.

The performance characteristics of a motor are determined by the materials used in its construction; therefore, the selection of appropriate materials is of critical importance [8,16,17]. Ferromagnetic materials employed in the stator of BLDC motors significantly influence the motor’s output parameters [16,18,19,20,21]. Variations in the design and type of these materials affect the motor’s performance, particularly its efficiency and torque output [16].

According to Nizam et al. [22], the use of ferrite materials in the stator core reduces core losses, while implementing a ferrite core in the stator slots yields higher voltages and lower current levels. Considering the torque generated by electric motors, ferrite materials are more suitable for stator slot applications in order to achieve improved motor performance [22]. Panchal et al. [8] reported that employing higher-grade magnetic materials increases motor efficiency. Changing the stator core material from M19 to Hiperco resulted in improved efficiency for low-power motors. In the literature, BLDC motors are most commonly manufactured using M19 material [23,24,25,26,27]. In the study conducted by Rajkumar et al. [26], four BLDC motor configurations were analyzed: laminated steel (M19) for both stator and rotor; composite material for both stator and rotor; laminated steel (M19) for the stator combined with soft magnetic composite (SMC) material for the rotor; and SMC material for the stator combined with laminated steel (M19) for the rotor. The results indicated that stator and rotor assemblies composed of laminated steel (M19) and SMC materials exhibit favorable thermal insulation and improved performance for electric vehicle applications.

The growing demand for lightweight and energy-efficient electric drives intensifies research on the use of alternative materials and hybrid structures in BLDC motors, especially in the context of additive manufacturing technologies. Traditional ferromagnetic cores remain the benchmark for high-efficiency designs, but their weight, cost, and manufacturing complexity limit their use in some emerging applications. This study addresses the need for a systematic evaluation of how replacing conventional electrical steels with polymers and hybrid structures affects the electromagnetic performance of BLDC motors.

In this study, a comparative FEMM analysis of six BLDC motor configurations with different stator and rotor materials was carried out. For each configuration, the distribution of magnetic flux density, the variation in the flux linkage with rotor position, the induced electromotive force (Back-EMF) waveforms, and the electromagnetic torque derived from co-energy were determined. The obtained results provide a deeper understanding of the trade-offs between electromagnetic performance, material properties, and technological constraints, while simultaneously highlighting both the advantages and limitations of polymers and hybrid solutions in BLDC motor design.

## 2. Materials and Methods

**Figure 1:**
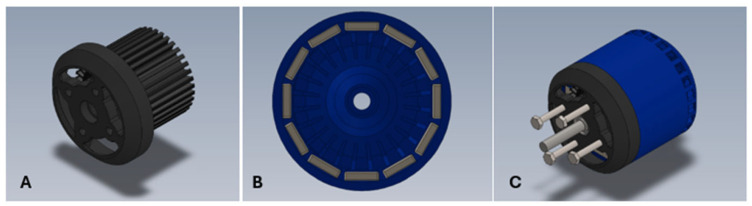
(**A**)—Stator model with mounting bracket—without windings, (**B**)—Front view of the rotor with permanent magnets arrangement, (**C**)—Complete assembly of the BLDC motor.

This study analyzes the influence of the type of ferromagnetic material on the operating characteristics of a brushless DC (BLDC) motor. For this purpose, an outrunner-type BLDC motor model was developed. Figure 1 presents the motor components and the complete assembly. The model included all main components: stator, rotor with magnets, bearings, and mounting elements such as the shaft and screws.

The analysis was carried out using the FEMM (Finite Element Method Magnetics) computational environment, designed for two-dimensional electromagnetic field analysis based on the finite element method. The modeled motor was of the outrunner type, in which the external rotor rotates around a stationary stator. The configuration featured twelve poles (six pole pairs) and a three-phase stator winding supplied with a sinusoidal current of 5 A amplitude. Each phase contained six turns. The rotor was equipped with NdFeB permanent magnets, grade N35. All mechanical dimensions, such as diameters, winding length, and air gap width, remained constant across all analyzed cases. Other structural elements, including the stainless-steel shaft, ABS stator mount, and copper windings, were also kept unchanged in all analysis variants.

Six different material configurations were analyzed, as summarized in Table 1. In the first, reference variant, a classic ferromagnetic configuration was used, with the stator core made of M19 electrical steel sheets and the rotor made of 1010 low-carbon steel. The second variant employed 1010 low-carbon steel for both the stator and rotor. In the third variant, the stator was made of magnetic PLA, while the rotor was made of ABS. In the fourth variant, both components were made of ABS. In the fifth variant, the stator was made of magnetic PLA with a metal sleeve, and the rotor of ABS. The last variant featured a stator made of ABS with a metal sleeve and a rotor made of ABS.

Simulations were conducted over the full rotor rotation range, from 0° to 360°, with a calculation step of 1°. In each computational iteration, the script automatically determined the corresponding electrical angle by multiplying the mechanical angle by the number of pole pairs, generated three sinusoidal currents phase-shifted by 120°, and applied them to the appropriate circuits in the FEMM model. The rotor was then rotated by one degree, and the magnetostatic solver computed the electromagnetic field for the new position. After each iteration, the magnetic flux in each phase, the co-energy of the magnetic field in the rotor region, and the electromagnetic torque were recorded. Based on the recorded flux values, the back-EMF waveforms were determined, and the electromagnetic torque was calculated.

## 3. Results and Discussion

**Figure 2:**
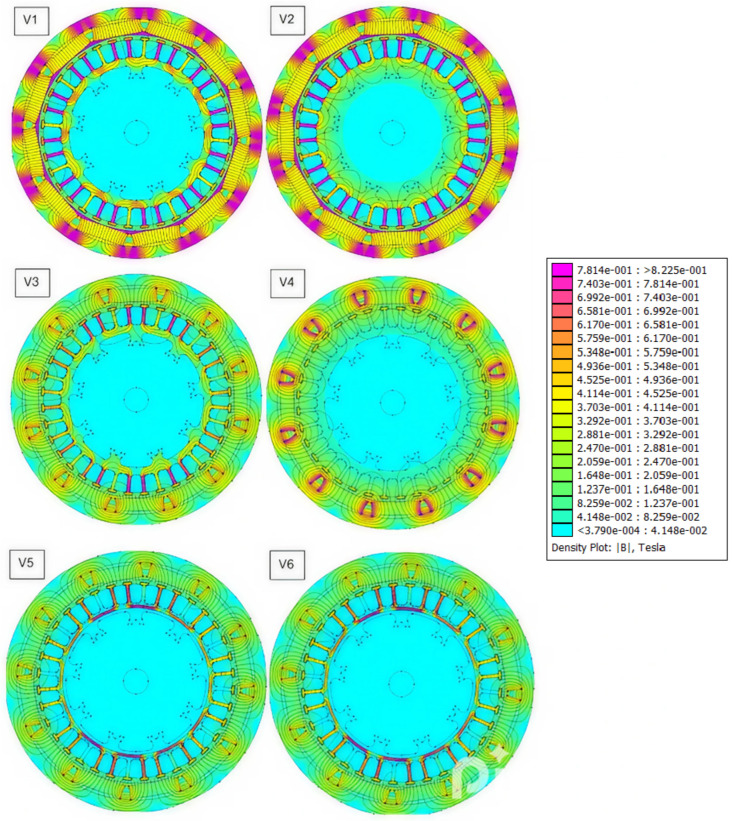
Example of magnetic flux density (B) distribution in the motor for all considered variants (V1–V6).

Figure 2 presents an example of the magnetic flux density distribution in the motor for all analyzed structural variants. In variant V1, the flux density map shows that the maximum induction in the stator teeth reaches approximately 1.83 T, which provides a safe margin below the saturation threshold of M19 steel (~2 T). In the rotor made of 1010 low-carbon steel, the magnetic induction falls within the range of 1.2–1.4 T, which helps to reduce hysteresis losses and allows for further design optimization (e.g., by in-creasing the number of turns or excitation current) without risking the loss of linear magnetic behavior.

For variant V2, the field distribution confirms that the induction in the stator teeth reaches about 1.84 T. Further current increases are predicted to cause flux waveform flattening and greater torque ripple. If the use of 1010 steel must be maintained for manufacturing reasons, a hybrid solution (e.g., M19 laminations in the teeth and 1010 steel in the yoke) is recommended, or structural modifications to reduce cogging torque—such as slightly skewed teeth or precise FOC control with d-axis current modulation.

Variant V3 confirms the above observations—the magnetic induction in the stator teeth is approximately 1.15 T, which is the upper limit for magnetic PLA. In this case, increasing the current does not increase the flux but leads to a sharp rise in hysteresis losses. The use of non-magnetic ABS in the rotor core results in no flux conduction, effectively “opening” the magnetic circuit and causing significant field dispersion into the air. As a side effect, however, cogging torque is significantly reduced—despite relatively high ripple compared to the average value, the absolute amplitude (0.28 Nm) is nearly ten times lower than in variant V1.

Analysis of variant V4 shows that the maximum induction (~0.78 T) occurs only in narrow regions near the N35 magnets. In the stator teeth made of ABS, the B field drops to 0.3–0.4 T, and in the rotor core, it is virtually zero (permeability ≈ 1).

In variant V5, the maximum flux density in the stator teeth reaches about 1.60 T, concentrated in narrow regions of increased field intensity. In the Magnetic PLA yoke, these values range from 0.6 to 0.8 T, while in the ABS rotor core, they fall below 0.3 T. Near the edges of the N35 magnets, localized peaks above 1.2 T were recorded, though the affected area remains very limited.

The final variant, V6, is characterized by field concentration in a narrow ring near the stator teeth, where B reaches 1.4–1.6 T. In the ABS yoke, induction drops to 0.5–0.6 T, and in the rotor core, it nearly vanishes. The metal sleeve helps capture part of the flux and limit its dispersion, but the absence of a ferromagnetic rotor still restricts the total magnetic permeance of the circuit.

**Figure 3:**
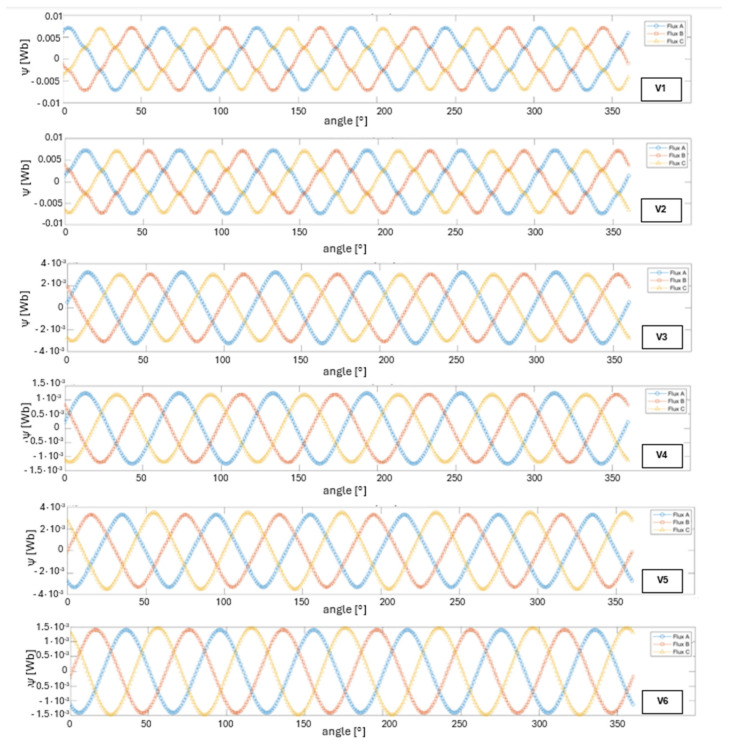
Flux linkage waveforms (Flux A, B, C) as a function of rotor angle for all analyzed cases (V1–V6).

Figure 3 shows the flux linkage waveforms (Flux A, B, C) as a function of rotor angle for all analyzed structural variants. In variant V1, the magnetic flux linkage in each phase reaches an amplitude of approximately 7.1 mWb, corresponding to a peak-to-peak value of 14.2 mWb. The waveform shape is nearly perfectly sinusoidal.

For variant V2, the flux amplitude is around 7.3 mWb (≈14.6 mWb pk–pk). The curves maintain their sinusoidal shape and 120° phase shift; however, compared to the M19/1010 configuration, slight flattening at the peaks is noticeable, caused by the lower magnetic permeability of 1010 steel.

Variant V3 is characterized by a flux amplitude of around 3.3 mWb (≈6.6 mWb pk–pk). The waveforms remain close to sinusoidal, but significant peak flattening indicates that the magnetic PLA material is approaching magnetic saturation. The maintained 120° phase shift confirms correct winding symmetry despite material limitations.

In variant V4, the flux amplitude is ±1.3 mWb (≈2.6 mWb pk–pk), which is a ~60% re-duction compared to the PLA/ABS configuration and about 80% compared to the steel-based variants. The waveforms retain a sinusoidal shape and 120° phase shift, though the absence of a ferromagnetic path significantly limits the field density in the air gap.

In variant V5, the flux amplitude is ±3.5 mWb (≈7.0 mWb pk–pk), representing a ~6% increase over the PLA/ABS configuration (3.3 mWb) and nearly three times the value obtained in the ABS/ABS setup. The waveforms remain clearly sinusoidal and phase-shifted by 120°, indicating that the use of a metal sleeve does not negatively affect magnetic field symmetry.

The final analyzed variant, V6, generates sinusoidal flux waveforms with 120° phase shift and an amplitude of ±1.5 mWb (≈3.0 mWb pk–pk). This represents an increase of approximately 15% compared to the full ABS configuration (±1.3 mWb), though it remains significantly lower than in the PLA/ABS variant.

**Figure 4:**
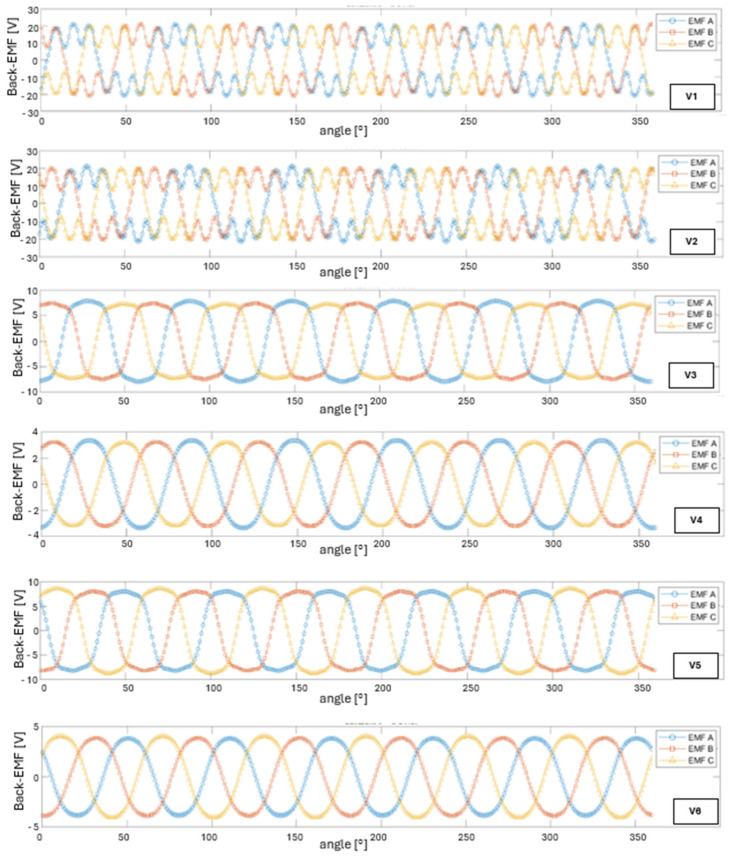
Back-EMF waveforms in phases A, B, and C for all analyzed configurations (V1–V6).

Figure 4 presents the back-EMF waveforms (phases A, B, and C) for all analyzed structural variants. In variant V1, peak values of ±19.5 V were recorded, which, at the given rotational speed, corresponds to a phase electromechanical constant of K_e_ ≈ 0.039 V·s/rad. When converted to the nominal speed reference, this yields approximately 4.1 V/krpm peak or 2.9 V/krpm RMS. The e(t) waveforms are nearly perfectly sinusoidal, with negligible higher harmonic content, indicating that low torque ripple can be expected under sinusoidal inverter supply.

For variant V2, the back-EMF peaks at ±20.1 V, corresponding to K_e_ ≈ 0.039 V·s/rad (≈ 4.2 V/krpm peak, 3.0 V/krpm RMS). The increase compared to variant V1 lies within model uncertainty, but it should be noted that 1010 steel introduces higher core losses, and the slightly higher voltage values result from increased flux in the yoke region.

Variant V3 shows back-EMF peak values ranging from ±7 to ±8 V (≈15 V pk–pk) at ω = 500 rad/s, which corresponds to K_e_ ≈ 0.015 V·s/rad (≈1.6 V/krpm peak, 1.1 V/krpm RMS). The slightly trapezoidal waveform shape results from the limited magnetic permeability of magnetic PLA and localized saturation in the stator teeth.

For variant V4, induced voltage peaks range from ±2.7 V to ±3.3 V (≈6 V pk–pk) at 500 rad/s. This corresponds to K_e_ ≈ 0.006 V·s/rad (≈0.63 V/krpm peak, 0.45 V/krpm RMS). The trapezoidal waveform shape results from the absence of a ferromagnetic path, causing the magnetic field lines to close almost entirely through air.

Variant V5 reaches peak back-EMF values of ±7.5 to ±8.5 V (≈16 V pk–pk), correspond-ing to K_e_ ≈ 0.016 V·s/rad (≈1.7 V/krpm peak, 1.2 V/krpm RMS). This is about 7% higher than in variant V3 and roughly 2.5 times greater than in variant V4. The waveforms exhibit a more sinusoidal shape compared to the PLA/ABS configuration, confirming that the use of a steel sleeve improves magnetic coupling.

**Figure 5:**
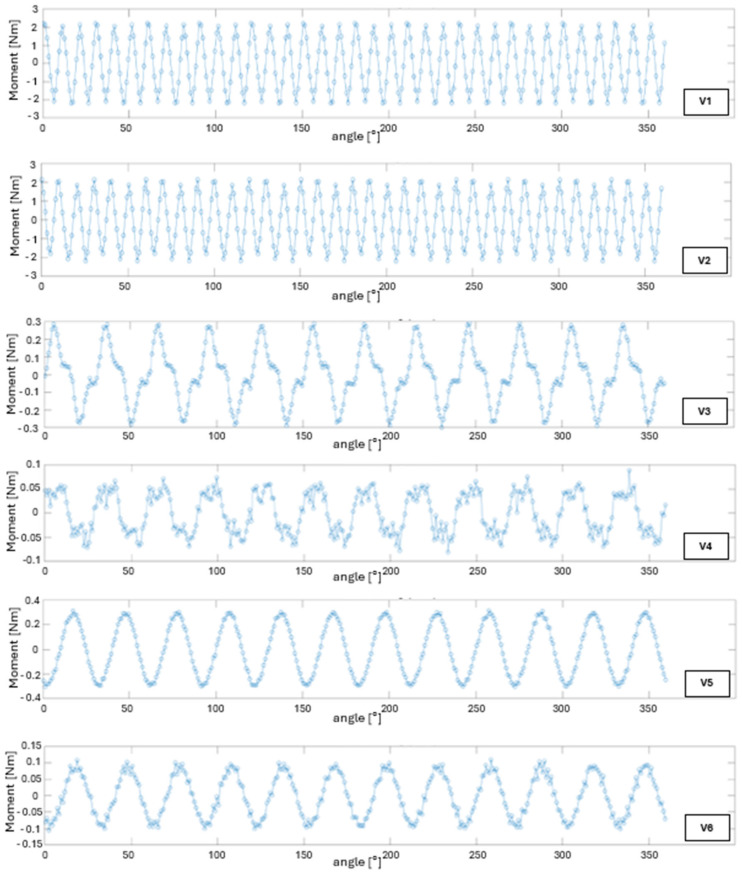
Torque waveform derived from co-energy (T) as a function of rotor angle for all analyzed cases (V1–V6).

In variant V6, back-EMF peaks range from ±3.6 V to ±4.0 V (≈7.6 V pk–pk), which corresponds to K_e_ ≈ 0.008 V·s/rad (≈0.84 V/krpm peak, 0.6 V/krpm RMS). Compared to the ABS/ABS configuration, this reflects an approximately 35% increase in voltage constant, further confirming the positive effect of the sleeve on enhancing magnetic coupling. Figure 5 presents the electromagnetic torque waveforms, determined using the co-energy method, as a function of rotor angle for all analyzed design variants. For variant V1, the peak instantaneous torque reaches approximately ±2.2 Nm (4.4 Nm pk–pk), with an average value over a full rotation of 1.44 Nm. Torque ripple is ±0.7 Nm, which accounts for less than 49% of the average torque. This moderate level of ripple confirms the beneficial effect of the 12-pole configuration and the appropriately selected air gap on cogging torque reduction.

In variant V2, the maximum torque is ±2.1 Nm (≈4.2 Nm pk–pk), with an average value of 1.30 Nm. The ripple remains at ±0.7 Nm, which, relative to the lower average torque, results in an increased relative ripple of about 54%. This phenomenon is a direct consequence of the lower magnetic permeability of 1010 steel, which reduces the material’s ability to smooth the air-gap flux distribution.

Variant V3 generates an electromagnetic torque with instantaneous values reaching only ±0.28 Nm (≈0.56 Nm pk–pk), and an average of approximately 0.05 Nm. This means the ripple exceeds 100% of the average value, resulting in noticeably non-uniform motor operation. The cause lies in the low magnetic permeability of PLA and the absence of a ferromagnetic rotor yoke, which, in variants V1 and V2, helped to smooth the flux distribution.

For variant V4, the instantaneous torque values reach ±0.07 Nm (≈0.14 Nm pk–pk), with an average of around 0.01 Nm. The ripple again exceeds 100% of the average, indicating that the motor produces a very low, but highly pulsating torque. In variant V5, the peak torque reaches ±0.30 Nm (≈0.60 Nm pk–pk), and the average is 0.06 Nm. This represents an increase of about 20% compared to variant V3 and nearly six times the value obtained in the ABS/ABS configuration. A reduction in torque ripple is also observed, indicating a more uniform magnetic permeance distribution in the stator.

Variant V6 is characterized by instantaneous torque values of ±0.10 Nm (≈0.20 Nm pk–pk) and an average of approximately 0.02 Nm, which is twice the value of the ABS/ABS variant, but still lower than that of the PLA/ABS configuration. Although the torque ripple exceeds 100% of the average, it is slightly lower than in the fully polymer-based design.

The simulation results demonstrate that stator and rotor material selection exerts primary control on the BLDC motor electromagnetic performance. A near-linear correlation is observed between the relative magnetic permeability of the core material and both flux linkage and Back-EMF: as permeability decreases (M19 → 1010 steel → magnetic PLA → ABS), flux linkage and induced voltage decline approximately proportionally. In contrast, torque ripple (ΔTpk/Tavg) exhibits a strongly nonlinear growth with decreasing permeability, increasing sharply for polymer-based variants and exceeding 500–700% in fully polymeric configurations (V3–V4). This behavior indicates that reduced permeability not only lowers average torque but also destabilizes torque production, which compromises the practical applicability of such designs in high-performance drives.

Quantitatively, the ferromagnetic configurations (V1–V2) produce peak flux density in the stator teeth around 1.8–1.9 T, which is well below the saturation limit of M19 laminations. This provides a safety margin for further optimization, for example, through higher current excitation or increased winding turns. The magnetic PLA variant (V3) shows tooth induction near 1.15 T, with localized saturation effects, while the ABS variants (V4) present very low air-gap flux (≈0.3–0.4 T in the teeth) and effectively open the magnetic circuit in the rotor (μr ≈ 1). Hybrid configurations with a thin 1010 steel sleeve (V5–V6) partially recover flux and voltage (V5: ≈56% of reference; V6: ≈20% of reference) and produce proportionally larger torque than fully polymer designs. Nevertheless, their absolute performance remains considerably lower than fully ferromagnetic machines due to the non-magnetic rotor limiting overall permeance.

From a design perspective, these findings emphasize that ferromagnetic cores remain indispensable in high-power-density and low-ripple applications. Nevertheless, polymer and hybrid cores may still be justified in specific cases, such as lightweight demonstrators, rapid prototyping, or applications where mass reduction and manufacturability are prioritized over efficiency. Hybrid strategies—including local ferromagnetic inserts, thin steel sleeves, or metallic caps on tooth tips—offer a practical compromise, improving magnetic coupling and reducing torque ripple in localized regions. The results further indicate that the reduction in performance in polymer-based configurations, while expected, is not the only relevant observation. The nearly linear dependence of flux linkage and Back-EMF on material permeability, combined with the exponential growth of torque ripple at low permeability, provides valuable insight into practical design trade-offs. This highlights that motor performance is governed not only by the absolute value of flux density but also by the continuity of the magnetic circuit ensured by ferromagnetic cores.

Solid ferromagnetic cores remain the unquestioned standard in high-performance electric motor design. A configuration using M19 laminations in the stator and low-carbon 1010 steel in the rotor provides a linked magnetic flux of approximately 7 mWb, an induced voltage (Back-EMF) of around 39 V peak-to-peak, and an average electromagnetic torque of about 1.44 Nm, with relatively low torque ripple of approximately 50% of the mean value (Table 2). Replacing the M19 laminations with 1010 steel in the stator results in only minor changes: a slight increase in flux and induced voltage is observed, while the electromagnetic torque decreases by less than 10%, and torque ripple rises to about 54%. It is worth noting that the core remains far from magnetic saturation, leaving room for further design optimization, for example, by increasing the current or reducing the air gap.

Replacing ferromagnetic materials with polymers significantly reduces the motor’s electromagnetic performance. In the variant with a stator made of magnetic PLA, the amplitude of the magnetic flux drops by half, the Back-EMF to about 38% of the reference value, and the average electromagnetic torque decreases to only 3.5% of the baseline value. Torque ripple increases sixfold, reaching approximately 560% of the mean value, indicating significant operational instability. In a motor made entirely of non-magnetic ABS, the parameters degrade even further: the linked magnetic flux reaches only 19% of the reference value, the induced voltage 15%, and the electromagnetic torque is nearly negligible. Torque ripple exceeds 700%, reflecting dominant pulsations and severely limiting the practical usefulness of such a design. These findings are consistent with experimental observations reported by Ellery et al. [28], who demonstrated that 3D-printed stator and rotor components made from magnetic polymer composites exhibit significantly reduced magnetic flux density and torque output due to low permeability and inter-layer discontinuities.

Introducing a thin 1010 steel sleeve into 3D-printed stators allows for partial recovery of lost parameters. In the variant with magnetic PLA and a steel sleeve, the flux amplitude increases to 56% of the reference value, Back-EMF to 41%, and the average torque exceeds 4%, representing nearly a 20% improvement over pure PLA and more than a sixfold enhancement compared to a fully polymer motor. Similar trends were observed in the ABS-with-sleeve variant, although absolute values remain lower; the average electromagnetic torque does not exceed 2% of the reference value. The presence of a non-magnetic ABS rotor limits the magnetic permeability of the entire circuit, and the steel sleeve effectively captures flux only in a narrow region near the stator teeth. Similar behavior was experimentally confirmed by Park [29] in studies on 3D-printed BLDC motors with metal stators, where the addition of thin steel sleeves or metal inserts partially restored the magnetic coupling and torque.

The analysis indicates a clear linear relationship between the magnetic permeability of the core and the values of the associated flux and back-EMF, accompanied by a dramatic increase in torque pulsations, defined as the ratio of ripple amplitude to average value. High-power-density motors with low torque ripple require the use of steel or laminated ferromagnetic cores. Printable materials, especially in fully polymer form, are primarily suitable for lightweight demonstrators, where minimizing mass, simplifying construction, and reducing cogging torque take priority at the expense of energy efficiency. Hybrid solutions, combining polymer with locally placed ferromagnetic material, offer a compromise between these properties, although their performance remains significantly lower than that of fully steel designs.

Further improvements can be achieved through advanced vector control techniques (FOC) with d-axis current modulation, the introduction of skewed tooth shaping, subtle magnet notching, as well as optimization of the air gap and precise placement of steel inserts in areas of highest magnetic flux concentration. Similar approaches for reducing torque ripple and electromagnetic vibration through geometric and material optimization have been reported by Dai et al. [30], confirming the effectiveness of such design strategies in permanent magnet BLDC and PMSM machines. A practical solution may also involve attaching thin metal caps to the tips of the stator teeth, which increases local magnetic permeability and more effectively suppresses electromagnetic torque pulsations.

## 4. Conclusions

The conducted study demonstrates the significant influence of core material on electric motor performance. Fully ferromagnetic designs, such as M19 laminated stators with 1010 steel rotors, achieve high flux linkage, substantial back-EMF, and a robust average torque with moderate torque ripple. Substituting ferromagnetic materials with polymers drastically reduces these key parameters while increasing torque pulsations, leading to unstable operation.

Hybrid solutions, incorporating thin 1010 steel sleeves in 3D-printed polymer stators, partially recover lost performance, with flux linkage, back-EMF, and average torque improving compared to fully polymeric motors. However, even these hybrids remain below fully metallic designs in absolute performance. The results reveal a clear relationship between core magnetic permeability and motor characteristics, confirming that polymeric motors are most suitable for lightweight demonstrators where low mass, simplicity, and reduced cogging torque are prioritized over efficiency. The comparative results demonstrate a nearly linear dependence of flux linkage and Back-EMF on core permeability, whereas torque ripple increases nonlinearly with reduced permeability, underscoring the necessity of ferromagnetic cores in high-performance BLDC motors

Further improvements could be achieved through advanced vector control strategies, air-gap optimization, slanted tooth design, and precise placement of ferromagnetic inserts to locally enhance permeability and suppress torque ripple. Such hybrid approaches offer a compromise between lightweight design and functional performance, though fully ferromagnetic cores remain the standard for high-power, low-ripple applications.

## Figures and Tables

**Table 1:** Material variants analyzed in the BLDC motor model.

Variant	Stator Core Material	Rotor Material
V1	M19 electrical steel sheets	1010 low-carbon steel
V2	1010 low-carbon steel	1010 low-carbon steel
V3	Magnetic PLA	ABS
V4	ABS	ABS
V5	Magnetic PLA with metal sleeve	ABS
V6	ABS with metal sleeve	ABS

**Table 2:** FEMM simulation results for different core variants. Torque ripple is defined as ΔT_pk_/T_avg_ × 100%.

	V1	V2	V3	V4	V5	V6
Flux amplitude Φ [mWb]	7.1 (100%)	7.3 (103%)	3.3 (47%)	1.3 (18%)	3.5 (49%)	1.5 (21%)
Back-EMF pk-pk [V]	39 (100%)	40 (103%)	15 (38%)	6.0 (15%)	16 (41%)	7.6 (20%)
Average torque T_avg_ [Nm]	1.44 (100%)	1.30 (90%)	0.05 (3.5%)	0.01 (0.7%)	0.06 (4.2%)	≈0.02 (<1.4%)
Torque ripple [%]	≈49	≈54	≈560	≈700	≈500	≈500

## Data Availability

The original contributions presented in the study are included in the article. Further inquiries can be directed to the corresponding author.
